# Four‐day plaque regrowth evaluation of a peptide chewing gum in a double‐blind randomized clinical trial

**DOI:** 10.1002/cre2.275

**Published:** 2019-12-14

**Authors:** Brian Kirkwood, Michael Miller, Jeffery Milleman, Kimberly Milleman, Kai Leung

**Affiliations:** ^1^ Dental and Craniofacial Trauma US Army Institute of Surgical Research San Antonio Texas; ^2^ ClinSmart LLC Newton Pennsylvania; ^3^ Salus Research, Inc. Fort Wayne Indiana

**Keywords:** biofilm(s), drug delivery, oral hygiene, salivary antimicrobial proteins

## Abstract

**Objective:**

Antimicrobial peptide, KSL‐W, formulated as an antiplaque chewing gum (APCG), was tested to evaluate the dental plaque inhibition activity and safety in an IRB approved and FDA regulated 4‐day plaque regrowth clinical study.

**Methods:**

This Phase 2 two‐armed placebo‐controlled, double blind, randomized (1:1), multiple dose, and single‐center study was evaluated in a proof of concept for the APCG containing 30 mg antimicrobial peptide KSL‐W. Twenty six generally healthy subjects were consented and randomized into the study. The subjects were administered a dose three times per day for four treatment days following a complete dental prophylaxis. Participants were prohibited from conducting oral hygiene care (teeth brushing, flossing, and/or mouth wash rinse) for the duration of the trial. Twelve to 16 hr prior to the baseline visit, the subjects were to abstain from oral hygiene care. The Quigley–Hein Turesky plaque index (QHT) score and the oral soft tissue clinical exams were obtained at both Day 0 and Day 4.

**Results:**

All randomized study subjects that received either APCG or placebo gum completed the study with no significant adverse events recorded. The APCG significantly inhibited the regrowth of dental plaque over the course of 4 days. The whole mouth data demonstrated a difference in the QHT between the APCG and the placebo gum of 1.14 (*SE* = 0.27) and 95% confidence bounds of 0.58, 1.70 with a two‐tailed *P* value of .0003.

**Conclusion:**

Considering the limited sample size, the proof of concept analysis in this Phase 2 study confirmed that APCG is effective against dental plaque formation and safe for human use. (ClinicalTrials.gov Study ID# NCT02864901).

## 1 INTRODUCTION

The gold standard for removing supragingival plaque are daily tooth brushing, flossing, and/or use of antimicrobial mouthwash(Haps, Slot, Berchier, & Vander‐Weijden, 2008). Inability for daily practice of good oral hygiene due to poverty, military deployment, or general lack of compliance are reasons for the development of an antimicrobial chewing gum that would assist with daily oral care and reduction of oral plaque.

For antimicrobials, the emergence of resistance to conventional antibiotics observed in oral pathogens strongly suggests the need to develop new and safe antimicrobial agents for the treatment and/or prevention of oral infections (Stein, 2005; Zhang & Falla, 2009). While more potent antibiotics exist, antimicrobial peptides exhibit pronounced cidal activity against some significant antibiotic‐resistant bacterial pathogens found in medical treatment facilities (e.g., *Pseudomonas aeruginosa*, *Acinetobacter baumannii*, methicillin‐resistant *Staphylococcus aureus*, and vancomycin‐resistant *Enterococci*) (Haney, Straus, & Hancock, 2019; Kumar et al., 2019). The mechanism that antimicrobial peptides is thought to kill bacteria occurs by disruption of microbial membranes via destabilization of the pore/channel formation or by exerting a detergent‐like action (Haney et al., 2019; Koczulla & Bals, 2003; Patrzykat & Douglas, 2005; Wiesner & Vilcinskas, 2010; Yount & Yeaman, 2005; Zasloff, 2002). In other cases, antimicrobial peptides can enter the cell without damaging the membranes (i.e., the nonmembrane effects of cationic peptides). Once within the cell, they may disturb intracellular bacterial functions such as DNA and/or protein synthesis or interfere with vital housekeeping cell functions such as chaperone‐assisted protein folding (Otvos et al., 2000; Patrzykat & Douglas, 2005). Further, some of these peptides also possess other biologic activities that can impact cell proliferation, immune induction, cytokine release, chemotaxis, and tissue repair (Elsbach, 2003; Koczulla & Bals, 2003).

Acquired resistance toward antimicrobial peptides is uncommon (Koczulla & Bals, 2003; Zasloff, 2002), though some bacterial strains are naturally resistant to certain antimicrobial peptides (Haney et al., 2019). Resistance mechanisms, if developed, may include reductions in transmembrane potential with a concomitant decrease in the attraction of cationic peptide antimicrobials (Yeaman, Bayer, Koo, Foss, & Sullam, 1998), sequestration of antimicrobial peptides by cell surface‐associated anionic exopolysaccharides (Friedrich, Scott, Karunaratne, Yan, & Hancock, 1999), microbial degradation of peptides (Guina, Yi, Wang, Hackett, & Miller, 2000), modification of the cytoplasmic membrane by a decrease in anionic phospholipid levels (Dorrer & Teuber, 1977), and efflux of peptide antimicrobials (Bengoechea & Skurnik, 2000). However, the fact that naturally occurring antimicrobial peptides have been conserved and remained functional throughout evolution is a strong testament to their efficacy and importance as effectors of innate defense (Haney et al., 2019; Yount & Yeaman, 2005).

The novel use of an antimicrobial peptide (KSL‐W) in a chewing gum formulation to help control plaque growth is the primary goal of this capability development that specifically targets service members deployed in austere environments (Leung et al., 2009). KSL‐W exhibits selective bactericidal activity against cariogenic bacteria (*Streptococcus mutans*, *Streptococcus* *sobrinus*, and *Lactobacillus acidophilus*) and early colonizers (*Actinomyces naeslundii*) (Na, Faraj, Capan, Leung, & DeLuca, 2007) but shows little effect on some members of the normal oral flora (*Streptococcus* *mitis* and *Streptococcus oralis* ; unpublished data). Further, per in vitro data, the peptide is degraded in gastrointestinal environments, therefore, suggesting it has no deleterious effect on the resident intestinal flora (Na et al., 2007). The use of chewing gum formulations also has beneficial effects on oral health by stimulating salivary flow and thereby promoting remineralization of tooth enamel. The peptide exhibited significant adsorption to hydroxyapatite (tooth‐like material), an important characteristic for an antiplaque agent (Faraj et al., 2007; Huang, Shi, Mao, & Gong, 2016; Na et al., 2007). In this proof of concept clinical study, we demonstrate that antimicrobial peptide KSL‐W is safe for human use and inhibits plaque regrowth in the absence of other oral hygiene methods.

## 2 METHODS

### 2.1 Trial design

**Figure 1:**
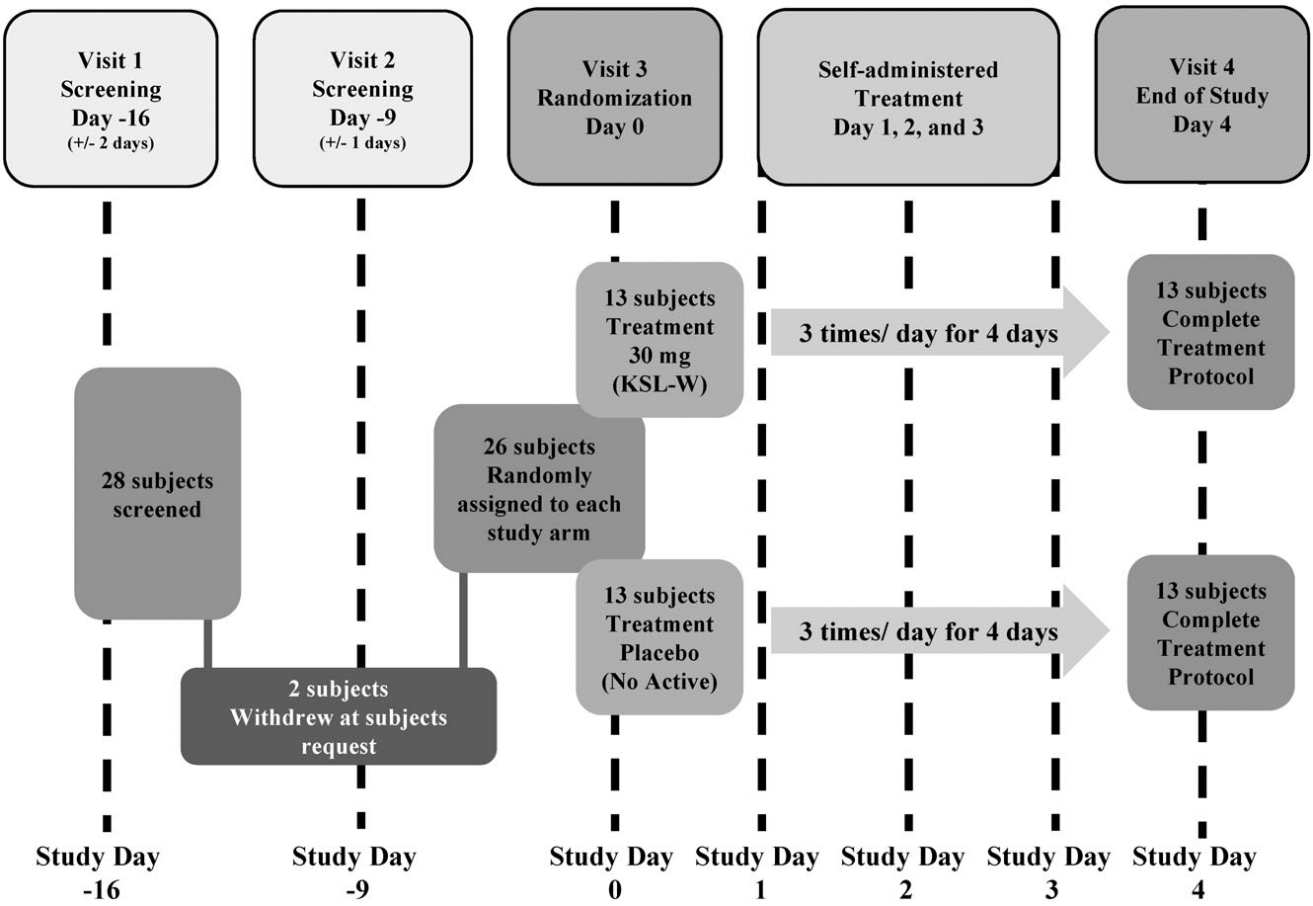
Schematic of the 4‐day plaque regrowth study design

**Figure 2:**
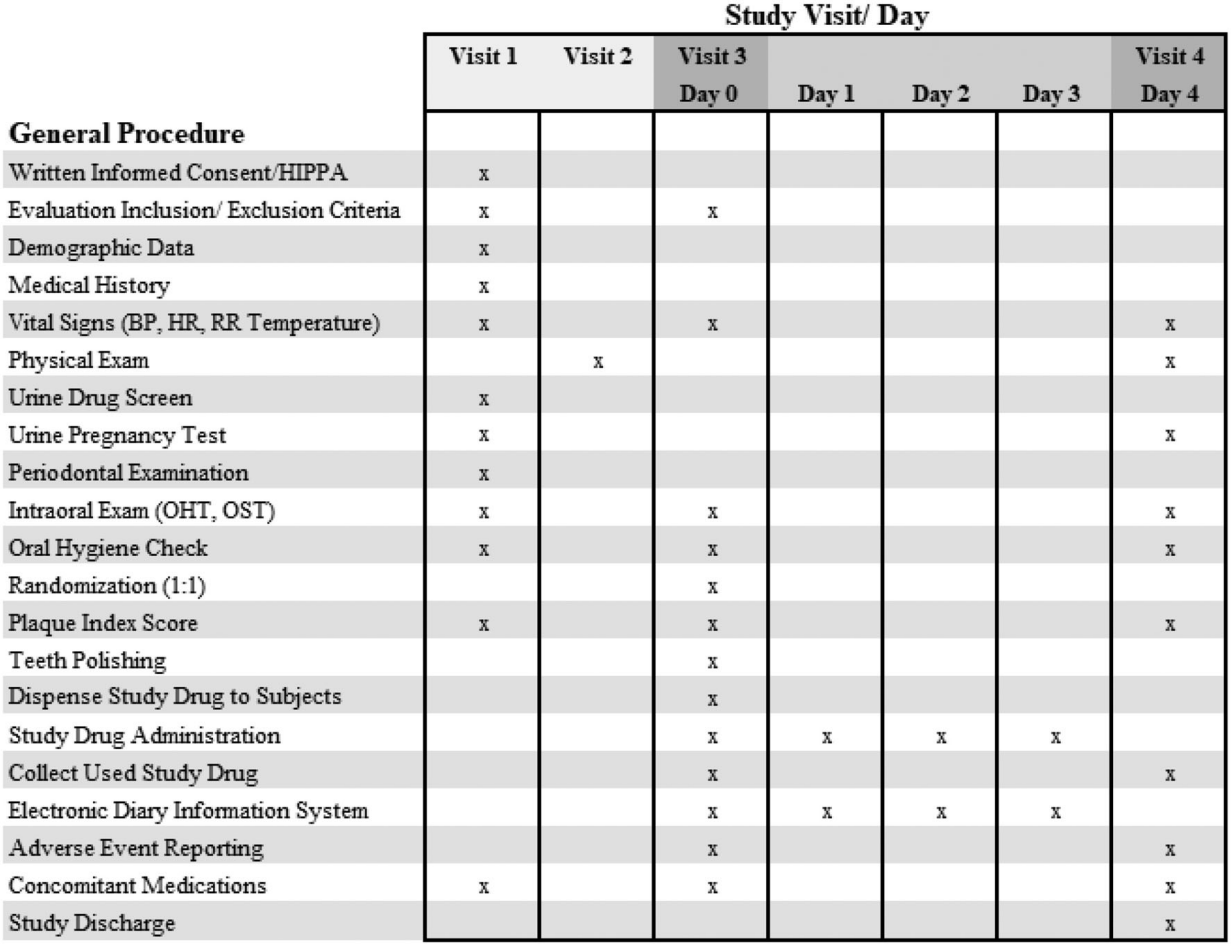
General procedures completed per visit over the course of the study

This Phase 2 two‐armed placebo‐controlled, double blind, randomized (1:1), multiple dose, single center study to evaluate the safety and proof of concept for an antiplaque chewing gum formulation (APCG) containing 30 mg antimicrobial peptide KSL‐W (Figure 1). Study subjects were administered a dose three times per day for four treatment days. Oral hygiene (teeth brushing, flossing, and/or mouth wash rinse) was prohibited throughout the trial, beginning 12 to 16 hr before both the screening and baseline (Day 0) visits, during the 4 days of treatment (Days 0, 1, 2, and 3) and ending after the periodontal examination and plaque assessment on Day 4 (Figure 2). The ability of antimicrobial peptide KSL‐W to reduce existing supragingival plaque was assessed. The oral soft tissue were examined. Changes from baseline, such as soft tissue erythema, ulceration, and sloughing were noted. The general procedures performed at each visit throughout the course of the study is summarized in Figure 2. Ethics approval was obtained from the US Investigational Review Board, Inc. (U.S.IRB2016SRI/06) located in Miami, FL 33143 on August 16, 2016 and the Human Research Protection Office, US Army Medical Research and Material Command (Ft. Detrick, MD). This clinical trial is available on https://clinicaltrials.gov/ (Study ID# NCT02864901). No changes were made to the trial design after commencement.

### 2.2 Participants, eligibility criteria, and setting

**Figure 3:**
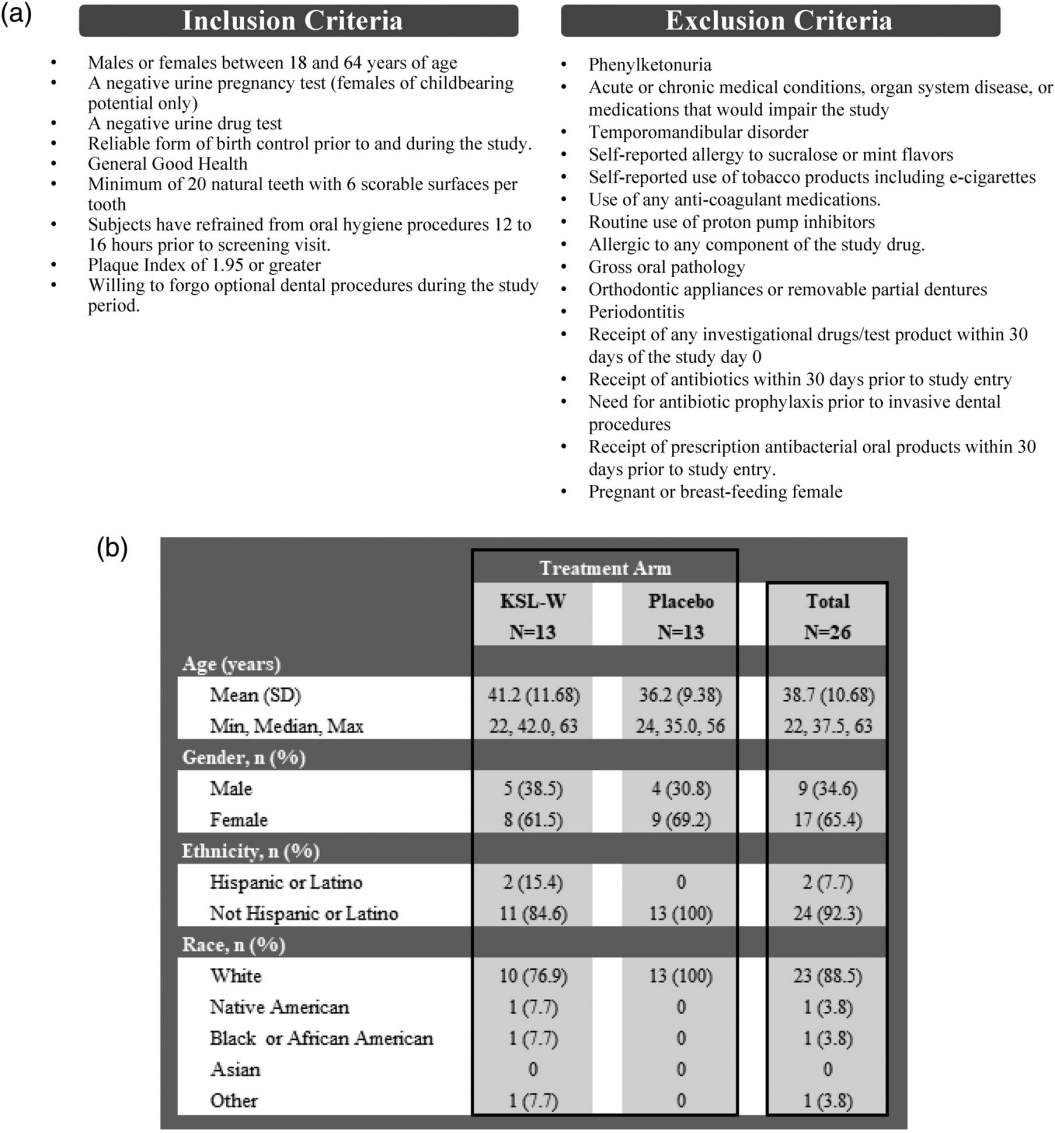
Study subjects that participated in the clinical trial. (a) Inclusion and exclusion criteria applied in the selection of the study subjects. (b) Study subjects demographics according to treatment arm

The study screened 28 subjects; of them, 26 subjects (9 males and 17 females; all females on contraceptives or postmenopausal) enrolled and completed the study. Subjects were generally healthy, without organ system diseases. Eligible subjects for this trial are required to meet all inclusion and exclusion criteria listed in Figure 3a. All subjects gave their informed consent prior to their inclusion in the study. Enrolled study subjects presented to the clinic (Salus Research Inc., Ft. Wayne, IN) on Day 0 (Visit 3) for the baseline plaque index score prior to dental prophylaxis treatment by a Registered Dental Hygienist. The first treatment dose was administered by the study staff, and the chewing was supervised at the clinic. Subject compliance was monitored through supervision of the first dose, controlled distribution of the product and collection of the product. For unsupervised dosing, each subject was provided with bags to collect the used product. Each bag contained the used product of one chewed gum. The bags (11 total) were labeled to record product usage. Subjects returned the used product bags to the research center on Day 4 (Visit 4). Subjects recorded the start and stop time of each chew on the electronic diary information system. On Day 4, the subjects returned to the clinic for another assessment to record the subject's plaque index score.

### 2.3 Plaque index score

Quigley–Hein Turesky (QHT) plaque index was used in this study for comparison of the two‐study arms and the different regions within the oral cavity (Turesky, Gilmore, & Glickman, 1970). The QHT plaque index is a 6‐point scale that ranges from 0 to 5 and scored as: (0) indicates no plaque present, (1) is for isolated flecks of plaque near the tooth cervical margin, (2) is for teeth that have a continuous band of plaque extending up to 1 mm from the gingival margin, (3) plaque extending beyond 1 mm up to a third of the tooth surface from the gingival margin, (4) plaque accumulation from one third to two thirds of the tooth surface, and (5) plaque accumulation more than two thirds of the tooth surface. The two evaluators used for this study were trained and calibrated to evaluate the QHT plaque index score. The same evaluator stayed with a given subject during Visits 3 and 4.

### 2.4 Test product and dose

Subjects received treatment with one of the following medications: APCG or a placebo chewing gum. The test product, APCG, contained the active ingredient, KSL‐W (US Patent No.: US 8,778,889 B2; Title: Antimicrobial decapeptide oral hygiene treatment), which is a cationic antimicrobial decapeptide. The antimicrobial peptide KSL‐W dose formulation of APCG used in this clinical trial was 30 mg (active). Inactive gum formulation (i.e., placebo) was a proprietary formula from Fertin Pharma A/S. Fertin Pharma A/S manufactured both placebo and test gum in accordance to cGMP.

### 2.5 Sample size justification

As a small proof of concept study, there was not an expectation of achieving a significant finding; hence, the sample size requirement was based on the precision necessary for designing a later phase study. A sample size of at least 12 subjects per study arm (antimicrobial peptide KSL‐W, Placebo) was determined to yield at least a posterior 94% chance of observing any antimicrobial peptide KSL‐W improvement in the sample mean QHT scores and at least an 81% posterior chance (given that an improvement was observed) that the projected sample size for a similar, later phase, confirmatory study, using a two‐tailed *p* = .05, power 90% criterion, is no more than 100 per study arm. These determinations were based on the results of an earlier Phase 2a trial (Tentative Title: A double‐blind, randomized, controlled, dose escalation clinical trial of an antiplaque chewing gum—Phase 2a safety, tolerability, and proof‐of‐concept in a gingivitis population; unpublished data).

### 2.6 Randomization

The random assignment of subjects to the two study arms was 1:1. The randomization schedule was generated by an independent biostatistician. Subject ID numbers were assigned in ascending numerical order as each subject signed the written informed consent form. Each subject who met the inclusion and exclusion criteria that were deemed fully eligible was assigned a unique randomized number. The randomization numbers were on the randomization list and were assigned to subjects sequentially prior to Visit 3.

### 2.7 Blinding

For this double‐blind study, the investigator, biostatistician, staff not involved in product preparation, and the study subjects were blinded to the treatment assignments throughout the duration of the study. The product preparation team received the randomization schedule from the unblinded independent biostatistician that was used to prepare the blinded product. The blinded product was labeled with a randomization number prior to being distributed to the research team and the study subjects.

### 2.8 Outcomes

#### 2.8.1 Safety

The primary objective was to access the safety and tolerability of antimicrobial peptide KSL‐W (30‐mg dose) delivered in a chewing gum formulation compared to placebo after multiple doses over a 4‐day treatment. Occurrence of local oral mucosal reactions, systemic reactions, and serious total body reactions were assessed. Physical examinations and vital signs were taken before treatment and at the end of the study.

#### 2.8.2 Proof of concept

The secondary objective was proof of concept by assessing the change in plaque regrowth from baseline in the whole‐mouth QHT of antimicrobial peptide KSL‐W (30‐mg dose) compared with placebo after multiple doses over a 4 day treatment. Subscores of the QHT were also summarized by computing average scores of selected subsets of measurements sites (interproximal, facial, lingual, maxillary, mandibular, and posterior).

### 2.9 Statistical analysis

The study evaluated the antimicrobial peptide KSL‐W, and placebo adverse event frequencies, incidences, and rate estimates were displayed side by side of affected body system, severity, and relationship to study drug. The estimated mean difference (KSL‐W minus placebo) in the change in plaque regrowth from baseline to Day 4 in the QHT and the estimated standard deviation of these reductions. The estimated means, standard deviations, or the total score and subscores of the QHT, visit (baseline, Day 4) and study arm (KSL‐W, placebo).

The primary proof of concept (efficacy) endpoint used the QHT whole‐mouth scores from the full analysis set and was based on the mean change from baseline to Day 4. The QHT whole‐mouth average was the average of scores only from surfaces evaluable for QHT, requiring that each subject have at least 20 teeth with six evaluable surfaces per tooth.

An analysis of covariance (ANCOVA) model was used to compare 30‐mg KSL‐W with placebo. The model included the baseline score and study arm (KSL‐W, placebo). Sensitivity assessments were employed to determine whether the effects of treatment on the slope of the baseline covariate and the effect of evaluator on the treatment placebo comparison can be ignored.

Estimates and 95% confidence bounds on all model parameters were summarized, and the estimated mean study arm difference from the final ANCOVA model was presented along with its standard error and 95% confidence bound. A two‐tailed *P* value for this difference was also presented. Secondary analyses using this model were performed on the subscores of the whole‐mouth QHT score.

The statistical analysis software utilized in these analyses was SAS, release 9.3.

## 3 RESULTS

### 3.1 Population demographics

This study enrolled 26 subjects with 13 subjects each assigned to the antimicrobial peptide KSL‐W treatment group and the placebo group. The study population had a mean age of 38.7 years with a standard deviation of 10.68 years and was predominately non‐Hispanic Caucasian woman. Neither the treatment nor the placebo group was a diverse population, but both groups were similar in age, ethnicity, and race. Differences in demographic variables between the two study arms were not significant. Data for population demographics are summarized in Figure 3b with the number of subjects listed and the corresponding percent or standard deviation reported in parenthesis.

### 3.2 Safety

All randomized subjects who received study medication completed the study and assessments. Safety issues did not result in the withdrawal of any subject. There were no deaths or other serious adverse events; no adverse events of any kind were reported during the study. Although no clinical laboratory tests were performed during the study, no clinically relevant findings were found from the physical examination or from vital signs at Visit 4/Day 4.

### 3.3 Compliance

One hundred percent compliance was noted in both treatment and placebo arms of the study. Each subject was required to administer a dose three times per day (morning/afternoon/night) during study Day 0 to study Day 3. Each dose event included a timed 20 min of chewing either APCG or the placebo.

### 3.4 Evaluator effect

**Figure 4:**
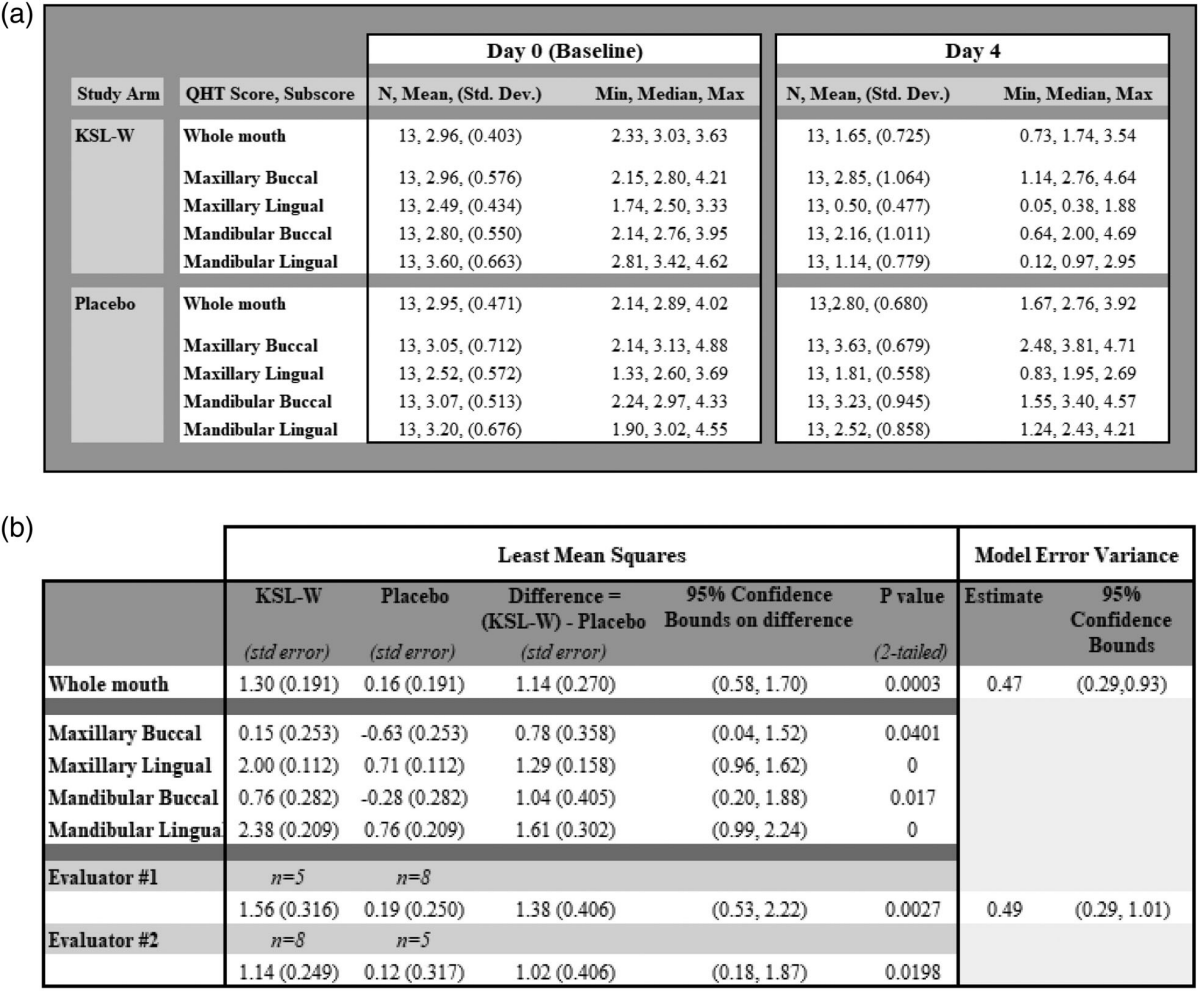
Quigley–Hein Turesky (QHT) plaque index scores for the whole mouth or subsections of the mouth. (a) QHT plaque index scores in each study arm for Day 0 (baseline) and Day 4. (b) The number represented in the first two columns is the plaque index score at baseline (Day 0) minus plaque index score at reevaluation (Day 4). Larger numbers represent a greater inhibition of plaque regrowth during the 4 days of the study

The two evaluators both had subjects that were in each study arm. Analysis was performed to establish the reliability of the QHT scores as recorded by each evaluators at Day 0 and Day 4. The evaluator effect was very small in comparison with other design effects, so the different evaluators in this study did not affect the study conclusions (Figure 4b).

### 3.5 Proof of concept

The proof of concept analysis examined the difference between baseline and study Day 4 whole mouth QHT scores by treatment arms (Figure 4a). The data indicate a clear difference in favor of antimicrobial peptide KSL‐W treatment, with a difference of 1.14 (*SE* = 0.27) and 95% confidence bounds of 0.58, 1.70 for the QHT score difference between treatment arms, with a two‐tailed *P* value of .0003.

Examination of the results for the difference between baseline and study Day 4 QHT (placebo treatment effect) for individual subjects showed that one subject in each treatment arm had QHT scores that were called into question for the assumption of normal distribution and linearity in the ANCOVA method used for the analysis. The data were reanalyzed by methods that were not based on these assumptions. Accelerated bootstrap analysis showed a 95% confidence interval of 0.51, 1.59 and indicating a shift to the left compared with the analysis specified in the Statistical Analysis Plan. The two sample, two‐tailed Wilcoxon *P* value was 0.0023. Therefore, this departure from the assumptions did not materially affect the conclusions of the study.

**Figure 5:**
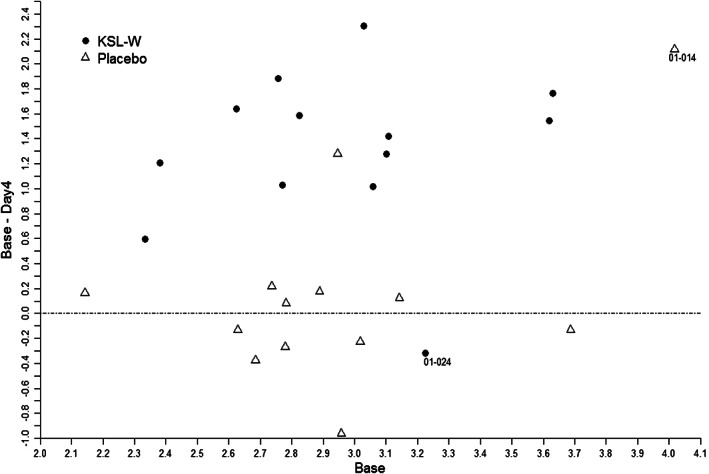
Data plot of each study subject per treatment arm. Comparison of the difference in the mean whole mouth plaque index score at baseline (Day 0) minus plaque index score at reevaluation (day 4) versus the mean whole mouth plaque index score at baseline (Day 0). Two outliers are represented by 01–014 and 01–024

Data for individual subjects' plaque regrowth scores are plotted in Figure 5. The plot shows that as a group, subjects in the antimicrobial peptide KSL‐W arm had greater differences than subjects in the placebo arm. Also, the two outlier subjects (01–014 and 01–024) are labeled in this plot (Figure 5).

Compared with placebo, the data for QHT score by quadrant demonstrated significant difference in favor of treatment with antimicrobial peptide KSL‐W. Data for QHT full mouth and subscores are summarized in Figure 4a,b.

## 4 DISCUSSION

The clinical significance is 30 mg of KSL‐W delivered as a chewing gum formulation minimizes plaque regrowth in the oral cavity. Efficacy was not the primary endpoint, and therefore, the study was not powered for determination of a statistically significant efficacy outcome. As a proof of concept study, the efficacy information from this study allows for a more traditional determination of sample size for a later study. The surprise result from this study is a statistically significant separation for the whole mouth QHT plaque index scores observed change on Day 4 from baseline. These results indeed satisfied the proof of concept for efficacy. The findings indicate that the required sample size for a future multicenter confirmatory study like this one would require substantially less than 100 subjects per group.

The decreased ability of dental plaque to regrow in the KSL‐W treatment arm suggests KSL‐W is upsetting the balance within the oral microbiome. Homeostasis within these microbial communities is regulated by various factors to include the host's innate immunity (Wiesner & Vilcinskas, 2010). The oral mucosal epithelia express small antimicrobial peptides such as alpha‐ and beta‐defensins, cathelicidins, and histatins. Human beta‐defensins (hBDs) specifically hBD‐2, is a prominent player in the regulation of the oral microbial environment. Unlike several other antimicrobial peptides, hBD‐2 are present in normal oral tissue even when inflammation is absent(Ghosh et al., 2018). Recent evidence suggests that *Fusobacterium nucleatum* induces expression of hBD‐2 from oral mucosal epithelia via a novel lipo‐protein, FAD‐I (Fusobacterium Associated Defensin Inducer) (Ghosh et al., 2018). However, other known opportunistic oral pathogens such as *Porphyromonas gingivalis* do not induce expression of hBD‐2. Consequently, *F. nucleatum* is resistant to the antimicrobial activity of hBD‐2, whereas *P. gingivalis* is not. These findings collectively indicate that antimicrobial peptides have an important role in regulating the oral microbial communities(Ghosh et al., 2018). An approach to bolster the innate immune defense against infection in the oral cavity is to deliver a synthetic antimicrobial peptide such as KSL‐W that is known to prevent colonization and disrupt oral biofilm formation (Concannon et al., 2003; Dixon, Jeffrey, Dubey, & Leung, 2009; Leung et al., 2005; Leung et al., 2009). The development of a chewing gum delivery mechanism for KSL‐W has previously been described (Faraj et al., 2007). This study shows that delivering KSL‐W via a gum formulation is safe and effective inhibiting dental plaque regrowth.

The purpose for development of this gum formulation is to reduce dental plaque accumulation in soldiers serving in austere environments. Simecek et al. (2014) demonstrated that dental caries‐related incidences contributes to 20–25% of dental disease nonbattle injuries, an alarming dental casualty rate, in deployed US Army personnel (Simecek et al., 2014). Dental caries is a demineralization of the tooth surface that occurs when cariogenic bacteria residing in the dental plaque (oral biofilms) metabolize sugar to produce an acidic environment (Balhaddad et al., 2019; Marsh, 2006). Dental plaque provides a protective environment that sustains growth of harmful pathogenic bacteria, including cariogenic bacteria. The gold standard to disrupt dental plaque formation is practicing good oral hygiene habits. Chewing gum is viable adjunct in maintaining good oral hygiene partly due to mechanical debridement and stimulation of salivary flow during gum chewing (Dawes, Tsang, & Suelzle, 2001; Hashiba, Takeuchi, Shimazaki, Takeshita, & Yamashita, 2015). Maintaining optimal oral hygiene is a challenge for soldiers deployed in austere environments, which contributes to an increase incidence of dental disease nonbattle injuries related to dental caries.

Currently, the soldiers have access to a xylitol chewing gum that is packaged in the Meal‐Ready‐Eat (MRE) meals and distributed to soldiers while deployed. In some situations, soldiers rely on the MRE for their source of food up to three times per day. Replacing xylitol chewing gum with the APCG capitalizes on these situations where the soldiers may not have the ability to perform optimal oral hygiene care. Xylitol is a nonfermentable sugar that works via a passive process where cariogenic bacteria is unable to metabolize it like other carbohydrates resulting in lowering the pH on tooth surface beneath the plaque (Lif Holgerson, Stecksen‐Blicks, Sjostrom, & Twetman, 2005; Syed, Chopra, Shrivastava, & Sachdev, 2016). However, MRE contains a large source of other carbohydrates to meet the energy requirements of the soldier while in combat. Therefore, fermentable sugars are abundantly available as the carbon source for cariogenic and plaque bacteria. Delivery of an antimicrobial peptide such as KSL‐W in these situations would add value because it actively strengthens oral innate immunity, prevents microbial colonization, and disrupts oral biofilm formation. Our study showed promising clinical results demonstrating that a chewing gum with an active ingredient, KSL‐W, inhibited dental plaque regrowth in the absence of other means of oral hygiene during the 4‐day test period. Preventing the regrowth of dental plaque removes the mechanism that cariogenic bacteria leverage to reduce pH on the surface of the tooth. These conditions mimic soldiers in austere environments who do not have the means to maintain proper oral hygiene.

The APCG is a valuable adjunct to routine oral hygiene for both the military population and the general population. A limitation in this study is a small homogenous population that is not representative of the general population. However, a focus of Phase 3 clinical trials will include a larger sample size that is a multicenter clinical trial of a representative general population. A consideration when analyzing a larger sample size is the potential of outliers similar to this study as we noted two outliers. There is a possibility that these two subjects did not report accurate responses on the study questioner. The self‐report questionnaire may not accurately indicate whether the subject followed protocol with abstaining from oral hygiene or chewing the gum according to protocol. Future studies will need to evaluate the effect of the APCG over an extended time course, and it would be interesting to evaluate this product compared with other chewing gum products where clinical trials have demonstrated dental plaque reduction. Literature searches failed to identify any other chewing gum products on the market or in development that are comparable with the conditions set in this clinical trial. Although xylitol gum is known to lead to dysbiosis within the dental plaque resulting in a reduction in caries (Syed et al., 2016), a KSL‐W containing gum is expected to perform better at dental plaque dysbiosis due to its antimicrobial activity and its ability to modulate the innate immune response (Semlali, Leung, Curt, & Rouabhia, 2011). The market potential of this chewing gum containing KSL‐W would impact multiple communities to include areas with limited access to dental care, long distance travelers, outdoor adventures, and the general population as a routine adjunct to current oral hygiene techniques.

## CONFLICT OF INTEREST

The authors declare no conflict of interest.
